# Identification of an Allelic Variant of the *CsOr* Gene Controlling Fruit Endocarp Color in Cucumber (*Cucumis sativus* L.) Using Genotyping-By-Sequencing (GBS) and Whole-Genome Sequencing

**DOI:** 10.3389/fpls.2021.802864

**Published:** 2021-12-22

**Authors:** D. S. Kishor, Hea-Young Lee, Hemasundar Alavilli, Chae-Rin You, Jeong-Gu Kim, Se-Young Lee, Byoung-Cheorl Kang, Kihwan Song

**Affiliations:** ^1^Department of Bioresources Engineering, Sejong University, Seoul, South Korea; ^2^National Academy of Agricultural Science, Rural Development Administration, Jeonju, South Korea; ^3^Department of Plant Science, Plant Genomics and Breeding Institute, College of Agriculture and Life Science, Seoul National University, Seoul, South Korea

**Keywords:** *CsOr* gene, orange endocarp, β-carotene, genotyping-by-sequencing, whole-genome sequencing, cucumber

## Abstract

The cucumber is a major vegetable crop around the world. Fruit flesh color is an important quality trait in cucumber and flesh color mainly depends on the relative content of β-carotene in the fruits. The β-carotene serves as a precursor of vitamin A, which has dietary benefits for human health. Cucumbers with orange flesh contain a higher amount of β-carotene than white fruit flesh. Therefore, development of orange-fleshed cucumber varieties is gaining attention for improved nutritional benefits. In this study, we performed genotyping-by-sequencing (GBS) based on genetic mapping and whole-genome sequencing to identify the orange endocarp color gene in the cucumber breeding line, CS-B. Genetic mapping, genetic sequencing, and genetic segregation analyses showed that a single recessive gene (*CsaV3_6G040750*) encodes a chaperone DnaJ protein (DnaJ) protein at the Cucumis sativus*(CsOr)* locus was responsible for the orange endocarp phenotype in the CS-B line. The *Or* gene harbored point mutations T13G and T17C in the first exon of the coding region, resulting in serine to alanine at position 13 and isoleucine to threonine at position 17, respectively. CS-B line displayed increased β-carotene content in the endocarp tissue, corresponding to elevated expression of *CsOr* gene at fruit developmental stages. Identifying novel missense mutations in the *CsOr* gene could provide new insights into the role of *Or* mechanism of action for orange fruit flesh in cucumber and serve as a valuable resource for developing β-carotene-rich cucumbers varieties with increased nutritional benefits.

## Introduction

Cucumber (*Cucumis sativus* L., 2*n* = 14) is a major vegetable crop in the *Cucurbitaceae* family with economic and nutritional importance. *Cucumis sativus* is native to India and was domesticated around 3,000 years ago and has extended its geographical distribution (Naegele and Wehner, 2017). Cucumbers are typically grown for fruits and are consumed fresh or as processed pickles across the globe. Cucumber fruit is ~96% of water and provides minerals, vitamins, protein, and a wide range of antioxidants (Butnariu and Butu, 2015). Cucumber ranked 3rd with an annual global production of 87,805,086 tons in an area of 2,231,402 ha (FAOSTAT, 2019) among the vegetable crops. China is the leading producer of cucumber, accounting for 70% of global production with an annual output of 54,315,900 tons, followed by Turkey, Iran, and Russia (Naegele and Wehner, 2017).

Most cucumber varieties have significant morphological differences in fruit appearance, size, color, and flavor (Che and Zhang, 2019). Cucumber fruit has exocarp, mesocarp, and endocarp tissue layers from the outer to innermost; fruit length varies from 5 to 40 cm with a round to cylindrical in shape (Che and Zhang, 2019). The mature exocarp has a green, white, or yellow skin color and mesocarp and endocarp are usually referred to as “fruit flesh.” The flesh color is an important quality trait that is directly associated with nutritional and economic benefits. The flesh color of the cucumber fruit mainly depends on the amount of β-carotene (Cuevas et al., 2010). The β-carotene serves as a precursor of vitamin A, which has health and nutritional benefits for humans and animals (Cazzonelli and Pogson, 2010; Nisar et al., 2015).

Genetic studies exhibited significant accumulation of carotenoid compounds, leading to orange- or yellow-colored fruit flesh in several germplasms of cucumber (Cuevas et al., 2010; Jack et al., 2011; Shen et al., 2011; Lu et al., 2015). In contrast, only a tiny amount of carotenoid compounds can be traceable with the white-colored endocarp in cucumbers (Waters et al., 2019). Carotenoid compounds are natural pigments formed *via* isoprenoid condensation and synthesized in many fruits, flowers, and vegetable crops (Nisar et al., 2015). Carotenoids appear yellow, orange, and red in plants (Cazzonelli and Pogson, 2010), which increase their nutritional value as well. Therefore, developing cucumber with orange or yellow fruit flesh could increase its nutritional and market value through introducing the value-added trait.

The first step in carotenoid biosynthesis begins with the plastid localized 2-C-methyl-D-erythritol 4-phosphate (MEP) pathway using glyceraldehyde-3-phosphate and pyruvate as primary substrates resulting in the synthesis of geranylgeranyl diphosphate (GGPP) (Supplementary Figure 1). This is followed by phytoene formation through condensation of geranylgeranyl diphosphate (CGDP) molecules *via* phytoene synthase (PSY) (Cazzonelli and Pogson, 2010). Further, phytoene desaturase (PDS) and ξ-carotene desaturase (ZDS) enzymes catalyze ξ-carotene by a series of 4 desaturation steps to produce lycopene from phytoene. In the next step, lycopene is cyclized to produce α-carotene and β-carotene by lycopene ε-cyclase (epsilon-cyclase) (ε-LCY) and lycopene β-cyclase (β-LCY), respectively. Hereafter, α-carotene and β-carotene are hydroxylated to form xanthophylls (Cazzonelli and Pogson, 2010; Nisar et al., 2015). To date, several genes and enzymes controlling carotenoid biosynthesis have been well documented in major crop plants (Bramley, 2002; Diretto et al., 2007; Welsch et al., 2008; Cazzonelli and Pogson, 2010; Qi et al., 2013). In contrast, recent reports emphasized that carotenoid content can also be controlled *via* regulation of chromoplast biogenesis by enabling a maximum rate of biosynthesis and accumulation capacity (Lu and Li, 2008; Cazzonelli and Pogson, 2010; Li and Yuan, 2013). The quantity of carotenoid accumulation is determined by the sequestration and storage capacity of synthesized molecules in a sink structure (Li and Yuan, 2013).

The *Or* gene in orange cauliflower encoding a protein associated with DnaJ cysteine-rich zinc finger domain induces the chromoplast formation from noncolored plastids due to single-locus mutation and confers a higher level of β-carotene accumulation in the tissues usually lacking carotenoids *via* chromoplast biogenesis (Lu et al., 2006). Identifying a mutation in the *Or* gene establishes a metabolic sink for β-carotene accumulation in the chromoplast of cauliflower and defines a key mechanism of plastid differentiation to control the accumulation of β-carotene in plants (Lu et al., 2006; Li and Van Eck, 2007; Lopez et al., 2008; Yang et al., 2011). In a recent study, the homolog of the cauliflower *Or* gene harbored a single mutation in melon, resulting in arginine to histidine amino acid substitution triggered β-carotene accumulation, entitled as a “golden” single nucleotide polymorphism (SNP) responsible for orange and nonorange fruit flesh trait in melon (Tzuri et al., 2015). In cucumber, the genetic mechanism controlling fruit flesh color has been reported. Molecular characterization of the Xishuangbanna group of cucumbers underlying orange endocarp fruit color showed single recessive gene inheritance of *CsaBCH1* (*Csa3G183920*), which further indicated a single SNP in conserved fatty acid hydroxylase domain of the β-carotene hydroxylase 1 (*BCH1*) protein responsible for the large amount of β-carotene content in the Xishuangbanna group (Qi et al., 2013). Besides, molecular mapping using F_2_ plants developed from a cross between yellow fruit flesh inbreed line PI200815 and white fruit flesh inbred line 931 showed that the single recessive gene “*yf* ” on chromosome 7 between the genetic distance of 0.6–0.3 centimorgan (cM) is responsible for yellow fruit flesh phenotype in cucumber (Lu et al., 2015). However, the molecular genetic mechanism of the *yf* gene has not been clearly understood.

Genetic studies in cucumber have confirmed that the orange/yellow color endocarp phenotype was controlled by a single recessive gene (Qi et al., 2013; Lu et al., 2015). The latest preprint revealed a novel mutation in the homolog of the orange cauliflower *Or* gene in cucumber, responsible for orange fruit flesh (Waters et al., 2019), and reported a new genetic resource for orange fruit flesh different from the Xishuangbanna group of cucumbers. However, this study has limited information on co-segregation of this mutation with the orange fruit flesh phenotype in a larger population (Waters et al., 2019). Therefore, the molecular genetic mechanism of the orange fruit flesh trait is uncertain; more studies are required on the allelic variation of the *CsOr* gene.

In this study, we report novel point mutations in the *CsOr* gene, responsible for an orange color endocarp in cucumber through genotyping-by-sequencing (GBS)-based genetic mapping and whole-genome sequencing. Genetic segregation analysis in a segregating population derived from a cross between white color fruit endocarp line CS-A and orange color fruit endocarp line CS-B showed that a recessive gene was responsible for the orange color endocarp. Expression of *CsOr* gene encoding DnaJ cysteine-rich zinc finger protein domain was induced in the CS-B line and β-carotene content accumulation increased accordingly in the endocarp tissue at fruit developmental stages. These results indicate that novel mutations in the *CsOr* gene are responsible for the orange color endocarp in the CS-B line, facilitating marker-assisted introgression of the *Or* allele and breeding cucumber varieties with higher nutritional benefits.

## Materials and Methods

### Plant Material and Endocarp Trait Evaluation

**Figure 1:**
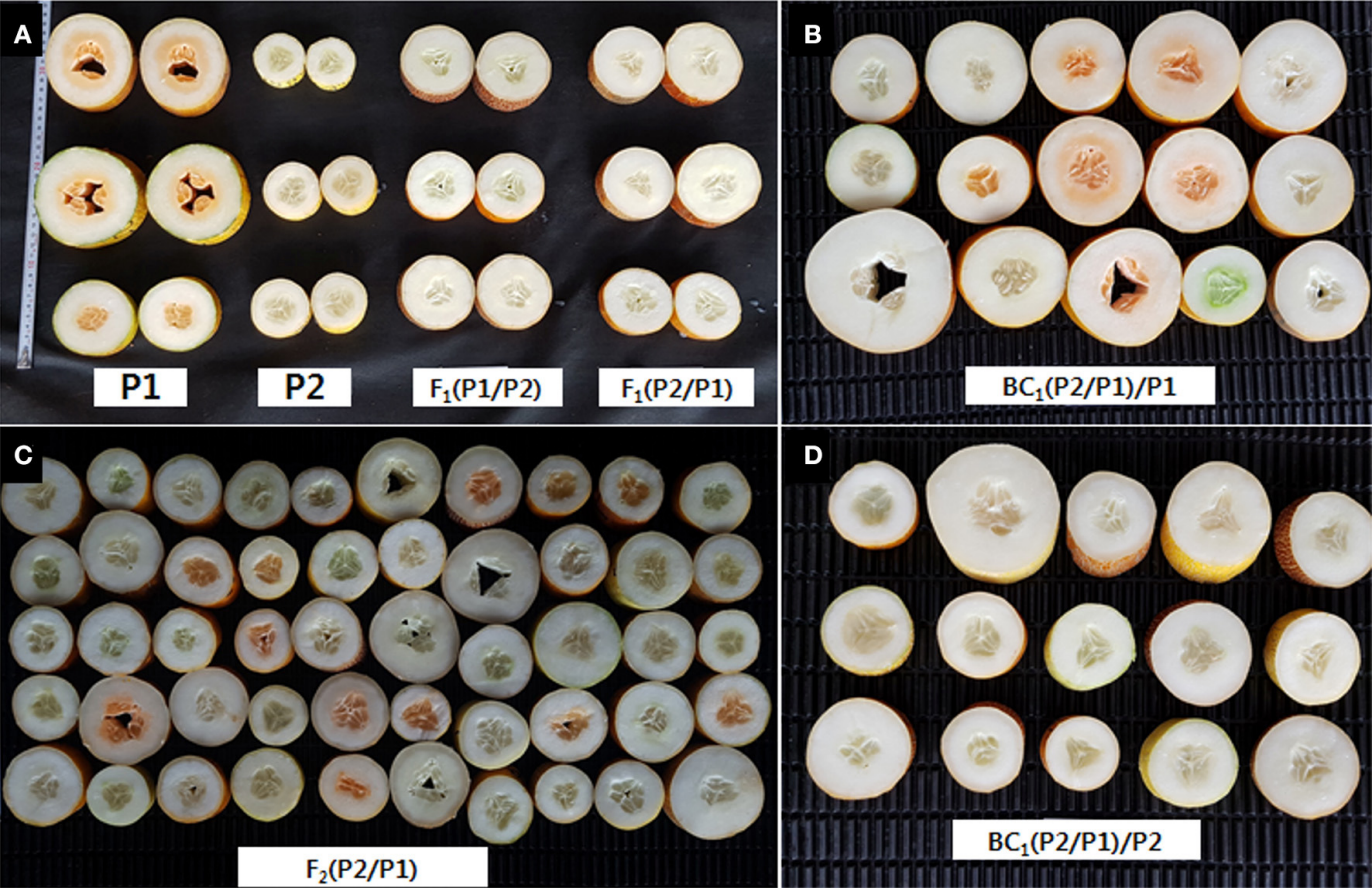
Phenotypic observation of fruit exocarp and endocarp color. **(A)** CS-B (P1), CS-A (P2), F_1_ (CS-B/CS-A), F_1_ (CS-A/CS-B). **(B)** BC_1_ (CS-A/CS-B)/CS-B. **(C)** F_2_ (CS-A/CS-B). **(D)** BC_1_ (CS-A/CS-B)/CS-A.

The semi-white type cucumber inbred line, CS-A (MEJ) (bred by Sejong University) and the orange endocarp cucumber breeding line, CS-B (selected from PI163217 through 3 cycles of self-pollination), were chosen as parents for developing an F_1_, BC_1_F_1_, and F_2_ populations (Figure 1). Seeds of PI163217 were collected from Agricultural Research Service (ARS), United States Department of Agriculture (USDA). The endocarp color of all the fruits from parents, F_1_, BC_1_F_1_, and F_2_ populations were evaluated at the experimental farm of Sejong University, Anseong, Republic of Korea, during the summer season of 2018. The chi-squared test was performed using Microsoft Excel 16.0 software to estimate segregation ratio of fruit endocarp color in F_2_ population derived from a cross between semi-white type cucumber inbred line, CS-A and orange endocarp cucumber breeding line, CS-B.

### Analysis of Carotenoid Content in Fruits

#### Sample Preparation

A total of three biological replicates of mesocarp and endocarp at 10, 20, 30, and 40 days after anthesis (DAA) from semi-white type cucumber inbred line, CS-A, and orange endocarp cucumber breeding line, CS-B were freeze dried and pulverized using liquid nitrogen (196°C) and stored in a deep freezer (−80°C) for extraction of the metabolites.

#### High-Performance Liquid Chromatography (HPLC) Analysis

The absolute content of β-carotene was estimated at 10, 20, 30, and 40 DAA from the endocarp tissue using a HPLC at the National Instrumentation Center for Environmental Management (NICEM), Seoul National University, with slight modifications for cucumber *via* saponification method (Larsen and Christensen, 2005).

For HPLC analysis, 1 g of each pulverized sample was thoroughly homogenized with 20 ml of 100% ethanol (EtOH) in a 50-ml Falcon tube. Each sample was washed with 100% acetone until sample becomes colorless and the resulting supernatant was filtered through a funnel and filter paper using a 500-ml conical flask. Further, exactly 15 ml of hexane and water higher than acetone volume were added to each sample extract. The sample extracts were shaken well and kept for 16 h. The upper hexane layer up to 5 ml was collected from each sample extract and transferred into new 50 ml glass test tubes. These test tubes were kept in nitrogen evaporator for 30 min to dry samples for further analysis. Finally, 1 ml of acetone was added to redissolve pigments in test tube and shaken well.

For saponification, a glass filter funnel was used to wash Ambersep 900 OH with water and was thoroughly dried using filter paper. The acetone extract was added to Ambersep 900 OH (1.3–1.4 g) and a stirring magnet in a centrifuge tube (30 ml) with a stopper. After stirring for 30 min, the solution was filtered through a 0.45-μm Minisart SRP25 Syringe Filter into a 4-ml brown *via*l for HPLC analysis. Lutein, zeaxanthin, α-carotene, β-carotene, and β-cryptoxanthin were used as external standards for peak identification and carotenoid analysis. All the standards were purchased from Kim and Friends (http://www.kimnfriends.co.kr/), Republic of Korea.

#### Liquid Chromatography-Diode Array Detector (LC-DAD)

The relative content of carotenoids was estimated at 40 DAA from both the mesocarp and endocarp tissues using the LC-DAD system at Functional Metabolomics Laboratory, Department of Systems Biotechnology, Konkuk University, South Korea (Ha et al., 2019), with slight modifications in cucumber.

For the LC-DAD analysis, 100 mg (dry weight) of each pulverized sample was thoroughly mixed with 3 ml of 100% EtOH containing 0.1% ascorbic acid in a 15-ml Falcon tube. Each sample was incubated at 85°C in a water bath for 5 min. Further, 120 μl of 80% potassium hydroxide (KOH) was added into the 15 ml Falcon tube and mixed thoroughly using a vortexer. Samples were incubated in a water bath for 10 min at 85°C and placed on ice for 5 min. β-Apo-8'-carotenal (100 μl, 25 ppm) was added as an internal standard (IS) with 1.5 ml of HPLC grade water. Further, each sample was extracted twice with 1.5 ml of hexane and centrifuged at 1,200 × g at 4°C for 5 min.

After centrifuge, 3 ml of upper aliquots were dried with nitrogen gas and redissolved with 50:50 (v/v) dichloromethane/methanol solution. Each extract was filtered using a syringe containing 0.2 μm polytetrafluoroethylene (PTFE) filter and relative peaks of carotenoids were obtained *via* the LC-DAD system and analyzed by comparing the standard and sample retention time. The IS used in this study was obtained from Sigma-Aldrich, St. Louis, Mosby, USA.

### Deoxyribonucleic Acid Extraction and GBS

Total genomic DNA (gDNA) was extracted from fresh leaf tissue of seedlings according to the cetyltrimethylammonium bromide (CTAB) protocol (Doyle and Doyle, 1986). The quality and quantity of gDNA were estimated using the NanoDrop 1000 (Thermo Fisher Scientific, Waltham, Massachusetts, USA). The gDNA samples were diluted to a DNA concentration of 100 ng/μl and stored at −20°C for further use.

Genotyping-by-sequencing libraries of parental lines (CS-A and CS-B) and 203 F_2_ plants were prepared and sequenced according to a recent protocol (Han et al., 2018). The high-molecular-weight gDNA samples were digested with *Mse*I and *EcoR*I restriction enzymes. The resulting DNA fragments were ligated to a pair of enzyme-specific adapters to generate a unique barcode for each sample. The DNA sample was then pooled and amplified *via* PCR to construct GBS libraries. The quality of GBS libraries was estimated using the Agilent 2100 Bioanalyzer System (Agilent Technologies, California, USA) and sequenced using the HiSeq 2000 Sequencing System (Illumina Incorporation, San Diego, California, USA).

### Single Nucleotide Polymorphism Calling and Genetic Mapping

Raw reads were demultiplexed based on the barcode sequence and quality trimming was performed to select the reads with a Phred score of 20 or above. The high-quality reads were then mapped to the “Chinese Long version 2” cucumber genome (Huang et al., 2009) using the Burrows-Wheeler Alignment (BWA) (Li and Durbin, 2010) and SNP calling was done using the Genome Analysis Toolkit (GATK) (McKenna et al., 2010). High-quality SNPs were filtered with a >60% SNP coverage, >0.05 minor allele frequency (MAF), and >0.08 inbreeding coefficient for further analysis.

For genetic mapping, GBS-based SNPs, fit the 1:2:1 ratio (*p* > 0.01), were selected based on the missing data in the parents (CS-B and CS-A) and SNPs appeared as a heterotype in the F_2_ plants. The linkage analysis was performed using CarthaGene software and genetic distance between markers in cM was estimated *via* the Kosambi mapping function. Quantitative trait locus (QTL) analysis was conducted according to the composite interval mapping (CIM) using Windows QTL Cartographer 2.5 (Wang, 2007).

### Whole-Genome Sequencing and SNP Discovery

Shotgun DNA libraries were prepared from high-quality gDNA of CS-A and CS-B using the TruSeq DNA-Seq Kit (Illumina Incorporation, San Diego, California, USA). Then, the libraries were sequenced for 250 cycles according to the guidelines of the manufacturer with the HiSeq 2000 Sequencing System (Illumina Incorporation, San Diego, California, USA) at the Phyzen, Republic of Korea. Quality trimming of raw reads was performed to discard reads with a Phred quality (Q) score of < 20 using the FastQC version 0.11.3 (http://www.bioinformatics.babraham.ac.uk/projects/fastqc/). Further, adapter sequences were removed using Trimmomatic (http://www.usadellab.org/cms/?page=trimmomatic). The high-quality reads were then mapped to the “Chinese Long version 3” cucumber genome (Li et al., 2019) using the BWA (Li and Durbin, 2010) and SNP calling was done using GATK (McKenna et al., 2010). Functional annotation of SNPs was performed using the SnpEff program (Cingolani et al., 2012). Gene information of cucumber carotenoid biosynthesis was obtained from the Kyoto Encyclopedia of Genes and Genomes (KEGG) pathway (https://www.genome.jp/kegg-bin/show_pathway?csv00906) and the National Center for Biotechnology Information (NCBI) protein database (https://www.ncbi.nlm.nih.gov/protein/).

### High-Resolution Melting (HRM) Analysis

High-resolution melting primers were designed using the Primer 3 (https://bioinfo.ut.ee/primer3-0.4.0/) (Supplementary Table 1). Genotyping of the F_2_ population was performed with the CsaOR-HRM1 primers using the following PCR conditions: initial denaturation at 95°C for 2 min, followed by 55 cycles of denaturation at 95°C for 20 s, annealing at 54°C for 20 s, and extension at 72°C for 30 s, and a final extension at 72°C for 1 min. The HRM analysis was carried out in a 20-μl reaction volume using the Rotor-Gene 6000 Thermocycler (Corbett, Australia). The chi-squared test was performed using Microsoft Excel 16.0 software to estimate cosegregation ratio of marker genotype in F_2_ population derived from a cross between semi-white type cucumber inbred line, CS-A and orange endocarp cucumber breeding line, CS-B.

### Multiple Sequence Alignment

Amino acid sequences of the *Or* gene from cucumber, melon, and cauliflower were downloaded from the NCBI protein database (https://www.ncbi.nlm.nih.gov/protein/). Multiple sequence alignment was performed with the Clustal Omega (https://www.ebi.ac.uk/Tools/msa/clustalo/). Editing and visualization were done with the Jalview 2.10.4 (http://www.jalview.org/).

### Quantitative Real-Time PCR

Total RNA was isolated from the mesocarp and endocarp fruit tissues of parental lines (CS-A and CS-B) at 10, 30, 40, and 50 DAA using GeneAll^®^ Ribospin Total RNA Purification Kit (Korea) according to the guidelines of the manufacturer. First-strand complementary DNAs (cDNAs) were synthesized using the Nanohelix Easy cDNA Synthesis Kit (Korea). Real-time PCR was performed using the CFX96 Real-Time PCR Detection System (Bio-Rad Laboratories, Hercules, California, USA) with the Toyobo Thunderbird SYBR qPCR Mix (Japan). Gene-specific primers for real-time PCR were designed with Primer3 (https://bioinfo.ut.ee/primer3-0.4.0/). The *CsActin* gene was used as an internal control. Primers used for real-time PCR are shown in Supplementary Table 2. Data were analyzed using the ΔΔCt method according to the previous studies (Livak and Schmittgen, 2001; Alavilli et al., 2018). The two-tailed *t*-test was performed to estimate the expression level between CS-A and CS-B parental lines.

## Results

### Inheritance of Orange Color Endocarp in Cucumber

The characterization of endocarp color in parental lines, CS-A and CS-B, shows white and orange endocarp, respectively (Figure 1A). Endocarp of the F_1_ derived from a cross between CS-B and CS-A and CS-A and CS-B was categorized as white (Figure 1A), while the fruits of BC_1_F_1_s derived from a cross between F_1_ (CS-A and CS-B) and CS-A demonstrated a white endocarp (Figure 1D). Nevertheless, BC_1_F_1_s obtained from a cross between F_1_ (CS-A and CS-B) and CS-B showed both the white and orange endocarp fruits (Figure 1B). Besides, F_2_ plants derived from a cross between CS-A and CS-B have white endocarp in 144 plants, whereas 62 plants showed the orange endocarp and segregated in a 3:1 ratio, suggesting that a single recessive gene was responsible for orange color endocarp phenotype in the CS-B line (Figure 1C and Table 1).

**Table 1:** Segregation analysis of fruit endocarp phenotype.

**Name**	**Population**	**Plant number**	**Fruit endocarp phenotype**				
			**White**	**Orange**	**Expected**	**χ2**	***p*** **Value^†^**
CS-A	P1	10	10	0	-	-	-
CS-B	P2	10	0	10	-	-	-
CS-B /CS-A	F_1_	6	6	0	-	-	-
CS-A/ CS-B	F_1_	7	7	0	-	-	-
CS-A / CS-B // CS-A	BC_1_F_1_	59	59	0	1:0	-	-
CS-A / CS-B // CS-B	BC_1_F_1_	61	32	29	1:1	0.15	0.70
CS-A / CS-B	F_2_	206	144	62	3:1	2.85	0.091

† *Not significant (p > 0.05)*.

### Determination of Carotenoids in Cucumber

**Figure 2:**
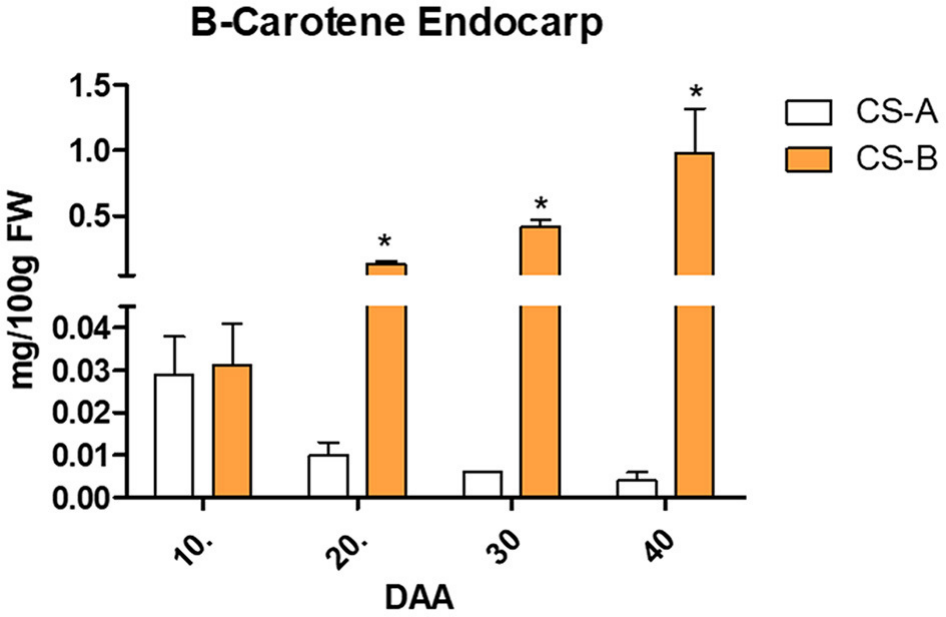
β-carotene content in endocarp of CS-A and CS-B lines. *indicate the level of significance (*p* < 0.05).

To quantify the β-carotene content in endocarp, tissue extracts of CS-A and CS-B lines were analyzed *via* HPLC system. Results showed that β-carotene contents were significantly increased during 20, 30, and 40 DAA in CS-B line (Figure 2). The HPLC analysis showed peaks analogous to standards (Supplementary Figure 2) and peaks analogous to biological samples of CS-A and CS-B (Supplementary Figure 3). Similarly, the relative content of the carotenoids in mesocarp and endocarp from tissue extracts of CS-A and CS-B lines was analyzed *via* the LC-DAD system. Results showed that lutein, zeaxanthin, β-cryptoxanthin, and α-carotene were significantly increased in the mesocarp tissue of the CS-B line compared with the CS-A line (Supplementary Figure 4). Similarly, α-carotene and β-carotene contents were also considerably increased in the endocarp of the CS-B line than the CS-A line (Supplementary Figure 4). Thus, the orange endocarp fruits had a significantly higher amount of β-carotene than white endocarp fruits. The LC-DAD analysis showed peaks analogous to standard and biological samples of CS-A (Supplementary Figure 5) and peaks analogous to standard and biological samples of CS-B (Supplementary Figure 6).

### Genotyping-By-Sequencing and Construction of Genetic Map *via* SNP-Based Linkage Mapping

Sequencing of GBS libraries yielded a total of 585.3 million reads totaling 59.12 Gbp (Table 2). Upon SNP calling, we generated 96,052 raw SNPs, which were subjected to further filtering. As a result, we identified a total of 67,848 filtered SNPs across the genome (Table 2). Additionally, SNPs with an SNP call rate of >0.3 were selected, which results in a total of 4,671 high-quality SNPs. Assuming a single gene inheritance for the phenotype, high-quality SNPs obtained from the GBS fit the 1:2:1 segregation ratio in F_2_ plants derived from a cross between CS-B and CS-A.

**Table 2:** Summary of genotyping-by-sequencing (GBS) data in the F_2_ mapping population.

**Description**	**Total**
Reads	585,373,907
Average read length (bp)	101
Length of read (Gb)	59.12
Raw SNPs	96,052
Filtered SNPs	67,848

**Figure 3:**
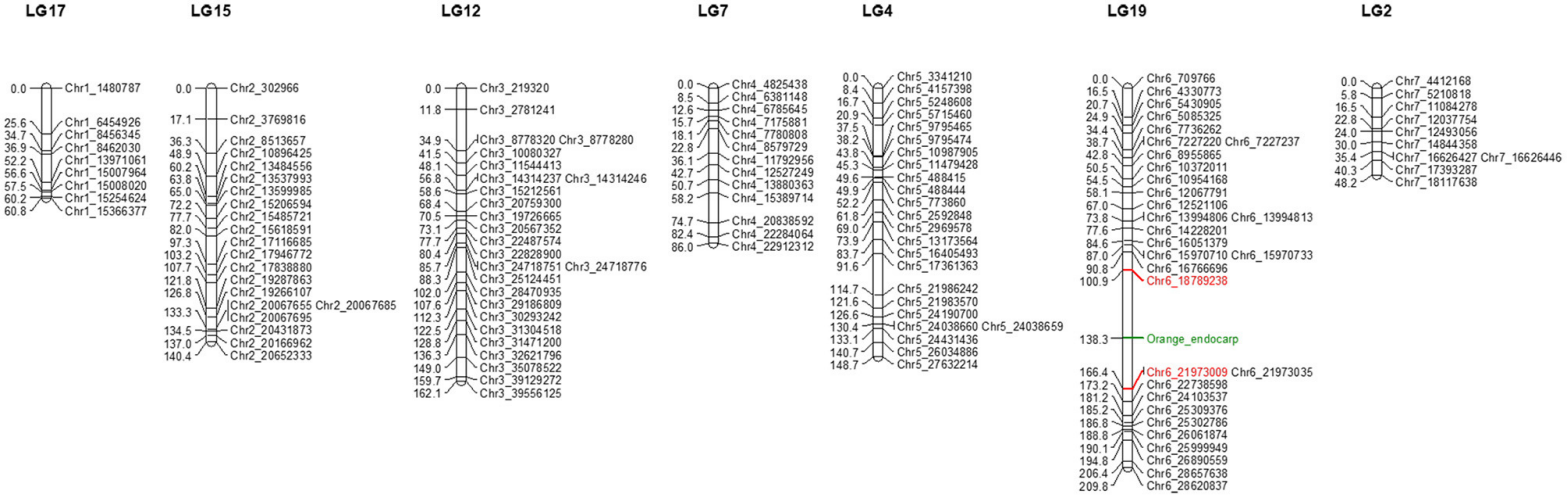
Linkage groups showing the distribution of high-quality single nucleotide polymorphism (SNP) markers on seven chromosomes of the cucumber F_2_ population. Marker names are given on the left side of the linkage group. Genetic distances between markers are indicated in centimorgan (cM). Loci associated with the orange color endocarp phenotype are indicated in green color between 100.9 and 166.4 cM on linkage group 19 (LG19).

Furthermore, SNPs missing in the parents (CS-B and CS-A lines) and SNPs that appeared as heterotype in the F_2_ plants were excluded from the high-quality SNPs dataset, which results in a total of 160 SNPs. In contrast, we used the resulting 160 (4.34%) SNPs and phenotype of endocarp from 206 F_2_ plants to infer genetic distance between the markers *via* the Kosambi mapping function for genetic map construction, resulting in a total of 19 linkage groups (Supplementary Table 3). Further, the gene responsible for the orange color endocarp phenotype was mapped on chromosome 6 at 138.3 cM of the 19th linkage group (LG) between Chr6_18789238 (100.9 cM) and Chr6_21973009 (166.4 cM) SNP markers (Figure 3). The distribution of 160 high-quality SNPs across the seven chromosomes of the cucumber genome in the F_2_ mapping population is shown in Figure 3.

### Prediction of Candidate Genes for the Orange Color Endocarp

We looked for candidate genes for orange color endocarp phenotype on chromosome 6 of the cucumber genome based on the genetic mapping results. Our BLAST results showed that two carotenoid-related genes such as *Csa6G448700* and *Csa6G452720* (*CsOr*) are located between 18.8 and 22 Mbp on chromosome 6. The gene *Csa6G448700* is located near 21.2 Mbp and encodes for carotene epsilon-monooxygenase, chloroplastic, which is generally known to be involved in the process of lutein biosynthesis (Tian et al., 2003; Fiore et al., 2006; Kim et al., 2009). However, the gene *Csa6G452720* (*CsOr*), detected near 21.6 Mbp, encodes for a DnaJ-like protein and known to be responsible for a higher level of β-carotene accumulation in orange cauliflower and melon (Lu et al., 2006; Tzuri et al., 2015).

### Genome-Wide Discovery of DNA Polymorphisms in Genes Associated With Carotenoid Biosynthesis *via* Genome Sequencing

We performed the high-throughput sequencing of the CS-A and CS-B cucumber lines and the resultant reads were mapped to the “Chinese Long version 3” genome (Li et al., 2019). A total of 116,265,512 and 117,086,894 reads were obtained for CS-A and CS-B, respectively (Table 3). Corresponding to each line, more than 12 GB read length and 70% of clean reads were generated upon preprocessing. Besides, mapping results show that CS-A and CS-B genomes were sequenced at a depth of 38.38X to 34.72X, respectively (Table 4). Analogous to the “Chinese Long version 3” reference genome, a total of 370,577 SNPs for CS-A and 951,815 SNPs for CS-B were detected (Table 4), which is distributed across the seven chromosomes in both the lines (Supplementary Table 4).

**Table 3:** Summary of sequencing for the cucumber varieties.

**Varieties**	**Raw reads**		**Trimmed reads**		
	**Reads**	**Read length (bp)**	**Reads**	**Read length (bp)**	**Coverage (%)**
CS-A	116,265,512	17,556,092,312	93,940,312	13,259,026,384	75.52
CS-B	117,086,894	17,680,120,994	90,417,082	12,710,158,922	71.89

**Table 4:** Mapping and single nucleotide polymorphisms (SNPs) information of the cucumber varieties.

**Reference variety**	**Cucumber varieties**	**Mapping data**				**SNPs**							
		**Reads**	**Mapped**	**Unmapped**	**Average**	**Non-**	**Synonymous**	**Intron**	**5'UTR**	**3'UTR**	**Intergenic**	**Others**	**Total**
			**reads**	**reads**	**depth**	**synonymous**							
Chinese long v3	CS-A	93,940,312	80,814,080	4,631,646	38.38	5,083	6,062	64,738	5,552	10,128	277,575	1,439	370,577
	CS-B	90,417,082	74,633,092	5,551,042	34.72	12,541	16,121	169,941	14,611	27,474	707,636	3,491	951,815

To detect functional genomic variation between orange and white-colored endocarp lines, we compared the sequencing information of the CS-B line with the CS-A line, “Chinese Long version 3” reference genome, and whole-genome sequencing data of two white-fleshed cucumber lines JEF and KWS available at the Department of Bioresources Engineering, Sejong University (unpublished data), yielding a total of 544,821 SNPs and 130,332 insertion-deletion polymorphisms (InDels). Further, comparative analysis of cucumber genome for carotenoid biosynthesis *via* the KEGG pathway and the NCBI protein database, based on the sequence variations in the genes associated with carotenoid biosynthesis, revealed that the CS-B line has 13 SNPs and 2 InDels in a total of seven genes related to carotenoid biosynthesis (Table 5). However, mapping to the KEGG pathway confirmed that only two candidate genes, such as *CsaV3_4G000740* and *CsaV3_6G040750*, play a direct role in the synthesis of pigments within the carotenoid biosynthesis pathway in CS-B breeding line, which suggested that DNA polymorphisms within the protein-coding sequence of these two candidate genes are associated with orange color endocarp phenotype in cucumber. Of these two candidate genes, we have identified a total of 3-point mutations at the position of 23,619,827 (T13G), 23,619,823 (T17C), and 23,619,716 (A124C) and an insertion of G at a position of 2,615,309 (A928GA) in the coding sequence of the *CsOr* gene (*CsaV3_6G040750*).

**Table 5:** Identification of candidate genes and sequence variation in CS-B for carotenoid biosynthesis.

**Chromosome**	**Gene Id**	**Position**	**Variation type**
1	*CsaV3_1G032340*	19,295,790	SNP
		19,296,096	SNP
		19,296,097	SNP
		19,297,612	SNP
2	*CsaV3_2G012080*	9,534,446	SNP
3	*CsaV3_4G000740*	421,165	SNP
		422,742	InDel (8 bp)
4	*CsaV3_4G007180*	4,916,062	SNP
		4,916,163	SNP
5	*CsaV3_4G017450*	10,889,227	SNP
6	*CsaV3_5G024720*	19,675,376	SNP
7	*CsaV3_6G040750*	23,615,309	InDel (1bp)
		23,619,716	SNP
		23,619,823	SNP
		23,619,827	SNP

### Single Nucleotide Polymorphisms Validation and Cosegregation Analysis

**Figure 4:**
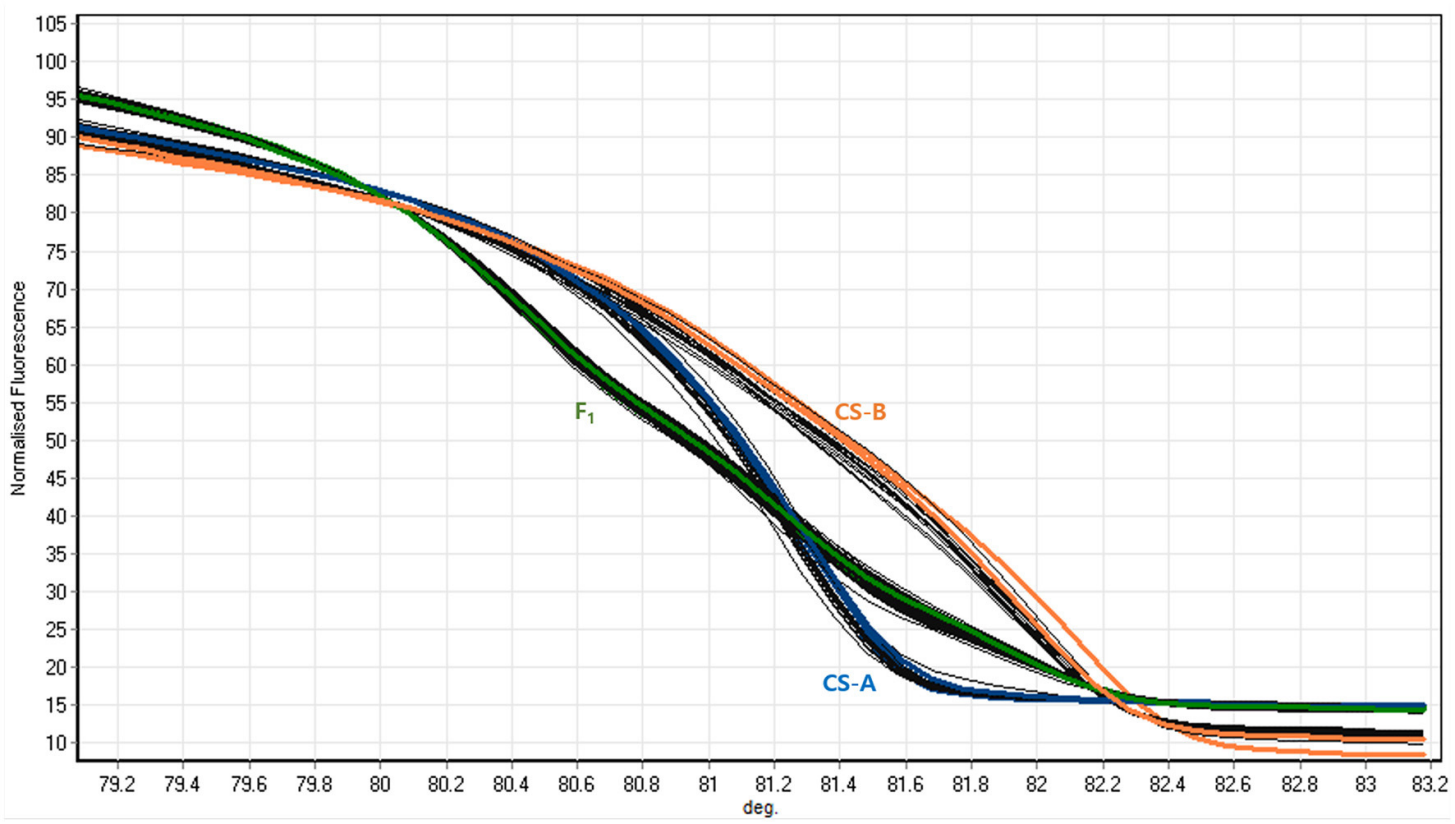
Melting curves generated for *CsaOr*-HRM1 marker. Curves of CS-A indicate CS-A genotype, F_1_ indicate heterozygous plants, and CS-B indicate CS-B genotype.

An HRM assay was conducted to determine the candidate gene responsible for orange color endocarp phenotype. Based on the sequence variations in *CsaV3_4G000740* and *CsaV3_6G040750* genes, a pair of HRM markers were prepared for each corresponding gene and validated to find cosegregation with the orange color endocarp phenotype in the F_2_ segregation population. Of these, CsaOR-HRM1 marker designed for the 2 SNPs located at the position of T13G (Ser13Ala) and T17C (Ile17Thr) on the first exon of *CsaV3_6G040750* (*CsOr*) gene (- strand) was able to distinguish CS-A and CS-B parental lines and heterozygous plants (Figure 4). Further, genotyping of the 195 F_2_ plants using CsaOR-HRM1 marker had an expected segregation ratio of 1:2:1 (Table 6), which revealed CsaOR-HRM1 marker genotype cosegregated with the orange color endocarp phenotype in F_2_ plants; none of the other SNPs among the candidate genes showed correlation with the orange color endocarp phenotype in F_2_ population (data not shown).

**Table 6:** Co-segregation analysis of fruit endocarp phenotype among 92 F_2_ plants derived from a cross between CS-A and CS-B.

**Cross**	**Number of F_**2**_ plants**	**White**	**Orange**	**H**	**ratio**	**χ2**	***p*** **Value^†^**
CS-A/CS-B	195	45	58	92	1:2:1	2.35	0.30

† *Not significant (p > 0.05)*.

### Sequence Analysis of *Or* Gene

**Figure 5:**
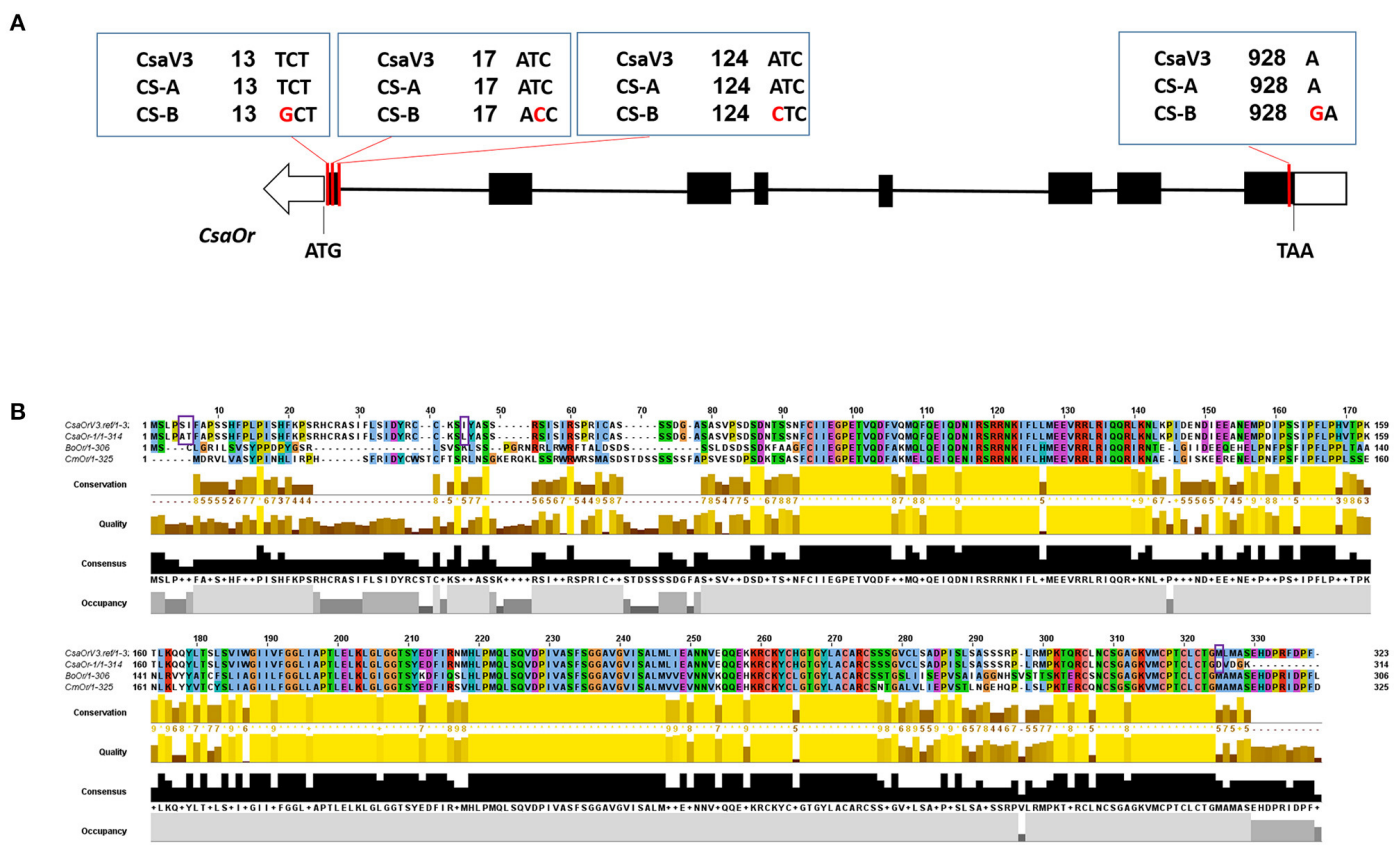
Gene structure of the *CsaOr* gene and multiple sequence alignment of *Or* protein. **(A)** Structure of the *CsaOr* gene, carrying the T13G, T17C, A124C point mutations, and an insertion of a “G” at position of 928 in CS-B line. **(B)** Multiple sequence alignment of *Or* protein from cucumber, melon, and cauliflower. *Or* protein homologs are represented with CsaOrV3.ref, cucumber reference sequence for *Or* protein from “Chinese Long v3”; CsaOr-1, Or protein from the *or* gene; *CmOr*, Or protein from *Cucumis melo* L.; *BoOr*, Or protein from *Brassica oleracea* L. Point mutations *i.e*., AA5, AA6, and AA45 and a frameshift mutation 325AA in the *or* gene are marked with squared purple line.

The cosegregation analysis showed that the T13G and T17C mutations have functional variation in the first exon of the *CsOr* gene on chromosome 6 (Figure 5A). These functional variations may play an essential role in β-carotene accumulation, thus causing orange color endocarp phenotype in CS-B breeding line. The *Or* gene encodes for DnaJ cysteine-rich zinc finger protein domain in plants (Lu et al., 2006). However, some researchers proposed the presence of an unidentified functional domain near the N-terminal region of the Or protein domain required for Or-PSY interaction (Yuan et al., 2015; Zhou et al., 2015c). In this study, multiple sequence alignment of *Or* amino acid sequences from the cucumber, sorghum, melon, citrus, cauliflower, and *Arabidopsis* revealed that CS-B breeding line carrying 3-point mutations at AA5, AA6, AA45 and a frameshift mutation at 325AA (Figure 5B), thus indicating the impact of SNPs in the gene coding sequence.

### Expression Analysis of CsOr Gene

**Figure 6:**
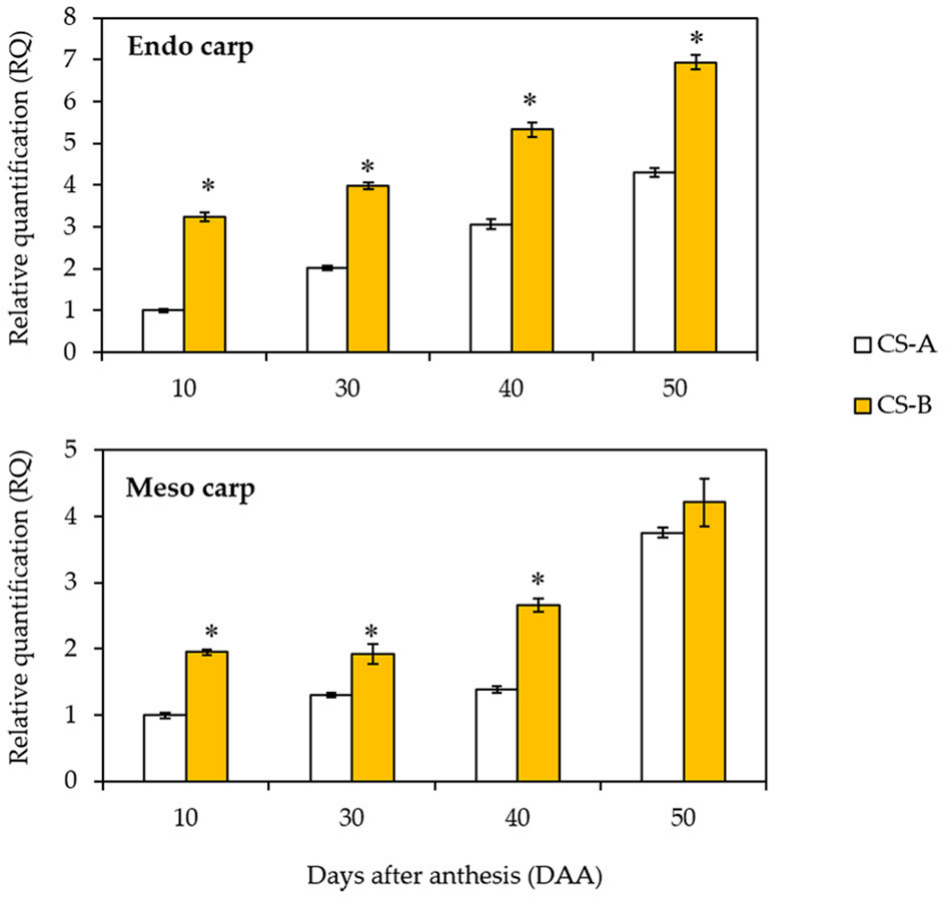
Expression of *CsOr* gene in CS-A and CS-B in different stages of fruit development. The *CsOr* gene expression levels were measured by quantitative real-time PCR using the total RNAs extracted from CS-A and CS-B cucumber fruit mesocarp and endocarp tissues at the designated days after anthesis. The *CsActin* gene was used as an internal control for normalization. For each experiment, the expression level of CS-A at 10 days after anthesis was used as a calibrator for quantification and was assumed as 1. Error bars represent SD of mean of 3 biological repeats. ^*^indicate statistical significance in least significant difference (LSD) test (*P* < 0.01).

We performed the expression analysis of *CsOr* gene to investigate the molecular basis of *Or* action in mesocarp and endocarp tissues at different stages of fruit development. Result showed that the *CsOr* expression is dramatically upregulated in endocarp tissue at fruit maturation stage (Figure 6). Though *CsOr* gene expression is found to be increased at fruit maturation stage, *CsOr* gene exhibited elevated expression in the mesocarp and endocarp of CS-B line at 10, 30, 40, and 50 DAA compared to the CS-A line. Furthermore, the elevated expression of *CsOr* gene from endocarp tissue is more evident at 30, 40, and 50 DAA in CS-B line (Figure 6), consistent with the orange fruit endocarp phenotype.

## Discussion

Identification and characterization of genes underlying carotenoid accumulation are essential to understand the molecular genetic mechanisms of orange color endocarp phenotype in fruit vegetable crops. To date, several genes controlling carotenoid biosynthesis have been studied in major vegetable crops, with an accumulation of carotenoids in the fruits (Ronen et al., 2000; Isaacson et al., 2002; Tadmor et al., 2005; Lu et al., 2006, 2015; Kato, 2012; Qi et al., 2013; Rodriguez-Concepcion and Stange, 2013; Tzuri et al., 2015). In cucumber, molecular mapping and identification of the *BCH1* gene revealed a key mutation, resulting in the orange phenotype in the Xishuangbanna group of cucumbers (Qi et al., 2013). The latest preprint of genetic analysis involving orange color cucumber proposed that the *BCH1* gene is not the only key genetic basis for orange color fruit flesh in cucumber; this study concluded that a frameshift mutation in the *CsOr* gene is likely causing orange coloration in cucumber lines (Waters et al., 2019). However, this study failed to provide detailed information on the SNP as a marker and its cosegregation with the orange color phenotype in a segregating population of cucumber. Therefore, understanding the genetic basis of the orange color phenotype with a robust experimental design would be necessary for identifying a gene and developing a molecular marker.

In this study, cucumber line CS-A with white endocarp was crossed with orange endocarp line CS-B and a segregation ratio of 3:1 was estimated in the F_2_ segregating population, suggesting that a single recessive gene is responsible for orange endocarp in the CS-B line. These results are inconsistent with the previous studies that indicate that a single recessive gene controlled orange/yellow color endocarp phenotype in cucumber (Bo et al., 2012; Lu et al., 2015). Further, the carotenoid analysis showed a significant increase of β-carotene content in the endocarp tissue during 20, 30, and 40 DAA in CS-B line, whereas relatively low content of β-carotene was found in the endocarp of CS-A inbred line; none of the other carotenoids were detected in both the CS-A and CS-B lines *via* the HPLC analysis. Additionally, efforts were made to quantify the relative content of carotenoids between the endocarp of CS-A and CS-B lines at 40 DAA using the LC-DAD system. The LC-DAD analysis showed a significant increase of lutein, zeaxanthin, β-cryptoxanthin, and α-carotene content in mesocarp and a substantial increase of α-carotene and β-carotene content in the endocarp of the CS-B breeding line (Supplementary Figure 4). Previous studies have also reported that cucumber lines with an orange endocarp phenotype exhibit a higher amount of β-carotene accumulation in the endocarp (Cuevas et al., 2010; Bo et al., 2012). Thus, the results of this study and previous studies suggest that a higher amount of β-carotene accumulation in the endocarp determining the orange color endocarp phenotype in cucumber.

Due to the rapid advent of next-generation sequencing (NGS), sequencing of plant genomes offers a large genome variation data set that can be used for linkage map construction in cucumber (Yang et al., 2012; Zhou et al., 2015a). Upon availability of complete genomic resources of the cucumber inbred line Gy14 and “Chinese Long” inbred line 9,930 (Huang et al., 2009; Yang et al., 2012), several genes underlying fruit color traits have been mapped and identified in cucumber (Bo et al., 2012; Qi et al., 2013; Yang et al., 2014a,b; Liu et al., 2015, 2016; Zhou et al., 2015b; Lun et al., 2016; Hao et al., 2018). Of these, the genetic basis for cucumber varieties with orange color endocarp phenotype was identified in the Xishuangbanna group of cucumber (Qi et al., 2013). In this study, genome-wide SNPs markers were used to map the gene responsible for orange color endocarp phenotype on chromosome 6 between 100.9 and 166.4 cM. Based on the initial mapping information, *Csa6G448700* and *Csa6G452720* (*CsOr*) genes were predicted to be in that genomic region on chromosome 6 and were likely associated with the carotenoids biosynthesis. These results provided the foundation for the verification of the candidate genes.

Whole-genome resequencing provided an efficient method for identifying genomic regions with millions of SNPs (Subburaj et al., 2019; Kishor et al., 2020a) and converting them into SNP-based markers (Kishor et al., 2019, 2020b, 2021). In this study, we performed the resequencing and analysis of CS-A and CS-B cucumber lines to explore genomic regions associated with carotenoid biosynthesis *via* mapping to the KEGG pathway. As a result, genomic variation within the *CsaV3_4G000740* and *CsaV3_6G040750* (*CsOr*) genes were predicted to be involved in the biosynthesis of carotenoids in CS-B breeding line. In the recent years, the identification of SNPs and the development of gene-based markers have gained importance to determine the genetic association with the phenotype in cucumber (Venkatesh et al., 2018). Therefore, SNPs detected within the candidate genes were exploited to develop gene-based HRM markers for SNP genotyping to determine the marker trait co-segregation in 195 F_2_ plants, derived from a cross between CS-A and CS-B lines. Genotyping with the HRM markers revealed an expected co-segregation ratio of 1:2:1 for T13G (Ser13Ala) and T17C (Ile17Thr) mutations on the first exon of *CsaV3_6G040750* (*CsOr*) gene (-strand), thus indicated a single recessive allele inheritance for orange color endocarp phenotype in cucumber.

Though a recent study detected insertion of G at a position of 2,615,309 (A928GA) in the coding sequence of the *Or* gene (Waters et al., 2019), however, this study revealed no correlation between the phenotype and genotype in the white and orange cucumber plants in the advance generation, thus applying this SNP marker was not reliable. Moreover, Waters et al. (2019) reported that genes encoding DNA chaperone protein had higher expression in the fruit flesh (mesocarp and endocarp) of colored fruits compared to the fruit flesh of white-colored fruits in F_2_ segregating populations of cucumber. By contrast, this study examined the expression of *CsOr* gene between the CS-A (semi-white type cucumber inbred line) and CS-B (the orange endocarp cucumber breeding line), which revealed that expression of *CsOr* gene was significantly higher in the endocarp of CS-B line than in CS-A line at 30, 40, and 50 DAA. These results suggest that β-carotene content in the endocarp tissue was increased by elevated expression of *CsOr* gene resulting in orange endocarp phenotype in CS-B line.

Previously, it was shown that *Or* allele encoding DNA chaperone protein induces chromoplast formation, resulting in the higher accumulation of β-carotene and exhibits orange endocarp phenotype in orange cauliflower and melon (Lu et al., 2006; Tzuri et al., 2015), which indicated that the *Or* gene is slightly sensitive to mutations within the coding region of the *Or* gene. Recent studies showed that genome editing of *Or* gene led to overexpression of mutated Or protein, resulting in the increased level of carotenoid accumulation in potato tuber, sweet potato, and rice callus (Lu et al., 2006; Kim et al., 2013; Endo et al., 2019). Thus, alteration in the expression level of *Or* gene *via* genome engineering might be useful to increase the carotenoid accumulation in cucumber. Besides, additional genetic factors showing that members of the Or family are associated with the major post-transcriptional regulation of PSY near the N-terminal region of the Or protein and promote the massive accumulation of carotenoids (Yuan et al., 2015; Zhou et al., 2015c; Osorio, 2019). In contrast, this study revealed that Ser13Ala and Ile17Thr substitutions are in the N-terminal region and located within the Or protein leader sequence. Therefore, we speculate that point mutations causing amino acid substitutions (Ser13Ala and Ile17Thr) near the N-terminal region could regulate PSY at post-transcriptional level and promote chromoplast formation, leading to massive accumulation of carotenoids in the CS-B line. However, further study is necessary to confirm the association of Ser13Ala and Ile17Thr substitutions with the regulation of chromoplast biogenesis in cucumber.

In conclusion, novel genetic variations identified in this study will facilitate the development of β-carotene-rich cucumbers varieties with increased nutritional benefits through marker-assisted introgression of the *Or* allele to the high-yielding cucumber varieties in breeding programs.

## Data Availability Statement

The datasets presented in this study can be found in online repositories. The names of the repository/repositories and accession number(s) can be found in the article/Supplementary Material.

## Author Contributions

KS conceptualized the study, chose the methodology and resources for this study, and acquired the funding. H-YL, DK, KS, HA, C-RU, and S-YL conducted formal analysis, chose the software for data analysis, and performed data visualization. DK curated the data, performed data validation, and wrote the first draft of the manuscript. KS, J-GK, and B-CK supervised the study. All the authors reviewed, edited, and approved the final version of the manuscript.

## Funding

This study was supported by the Cooperative Research Program for National Agricultural Genome Program (Project No. PJ01343201) Rural Development Administration, Korea and the Korea Institute of Planning and Evaluation for Technology in Food, Agriculture, Forestry, and Fisheries (IPET) funded by the Ministry of Agriculture, Food, and Rural Affairs (MAFRA) (Project No. 821009-03).

## Conflict of Interest

The authors declare that the research was conducted in the absence of any commercial or financial relationships that could be construed as a potential conflict of interest.

## Publisher's Note

All claims expressed in this article are solely those of the authors and do not necessarily represent those of their affiliated organizations, or those of the publisher, the editors and the reviewers. Any product that may be evaluated in this article, or claim that may be made by its manufacturer, is not guaranteed or endorsed by the publisher.
