# Supplementary material for: Identification of an Allelic Variant of the CsOr Gene Controlling Fruit Endocarp Color in Cucumber (Cucumis sativus L.) Using Genotyping-By-Sequencing (GBS) and Whole-Genome Sequencing
Source: Front Plant Sci. 2021 Dec 22;12:802864. doi: 10.3389/fpls.2021.802864 (PMC8729256; doi:10.3389/fpls.2021.802864)
Supplement: Supplementary file 1 [file Data_Sheet_1.pdf]

Identification of an allelic variant of the *Or* gene controlling fruit endocarp color in cucumber (*Cucumis sativus* L.) using genotyping-by-sequencing (GBS) and whole-genome sequencing

Supplementary Material

1 Supplementary Figures and Tables

1.1 Supplementary Figures

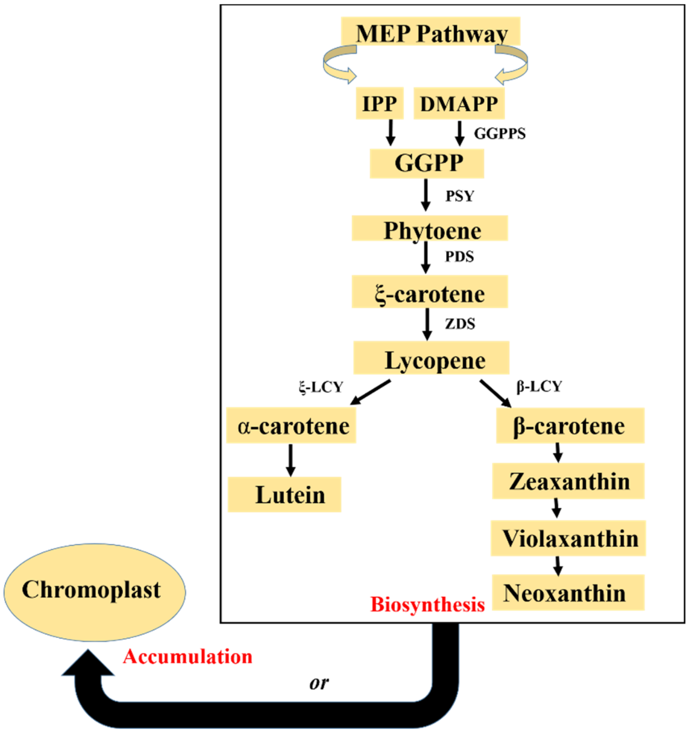

Supplementary Figure 1. Schematic model of carotenoid biosynthesis and accumulation pathway. Accumulation of carotenoids is determined by the rate of biosynthesis, degradation and storage capacity in a sink structure.

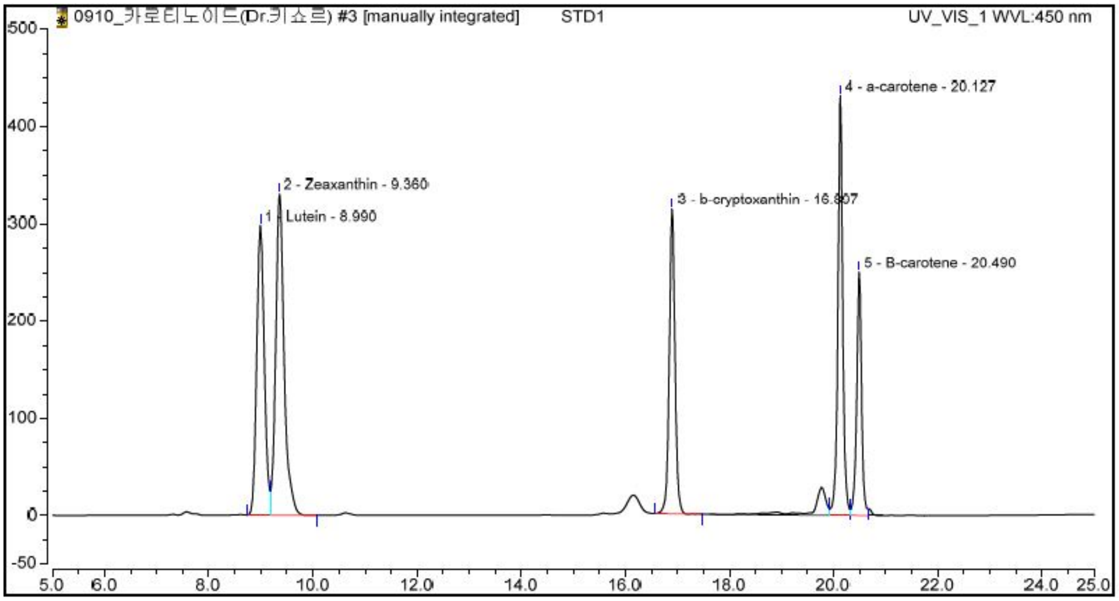

Supplementary Figure 2. HPLC spectrum of carotenoids for standards.

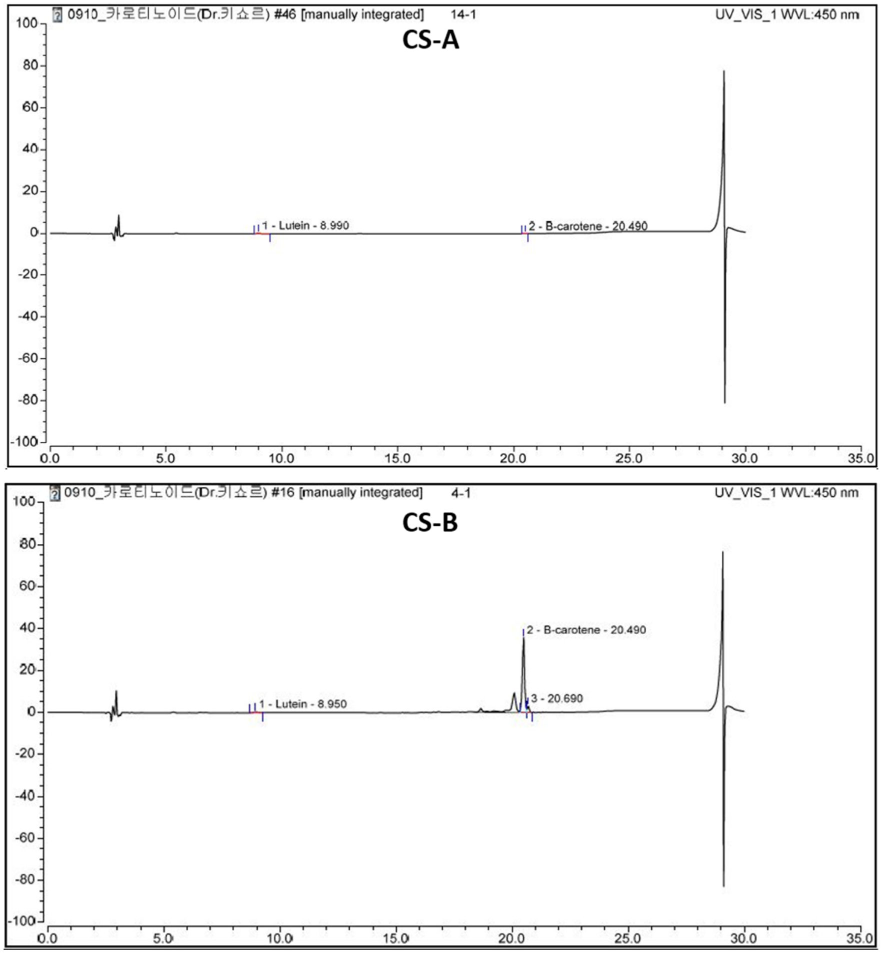

Supplementary Figure 3. HPLC spectrum of carotenoids in samples.

0.0 1.0 4.0

Mesocarp		Endocarp		Carotenoids
CS-A	CS-B	CS-A	CS-B	
0.04	3.04 *	0.17	0.38	Lutein
-	1.78 *	0.81	0.97	Zeaxanthin
-	0.91 *	0.81	1.57	β-Cryptoxanthin
0.02	0.26 *	0.23	4.36 *	α-Carotene
0.10	0.27	0.20	4.37 *	β-Carotene

Supplementary Figure 4. Heatmap representation for the relative content of different carotenoids in the fruits of CS-A and CS-B inbred lines. * indicate the level of significance ($P < 0.05$).

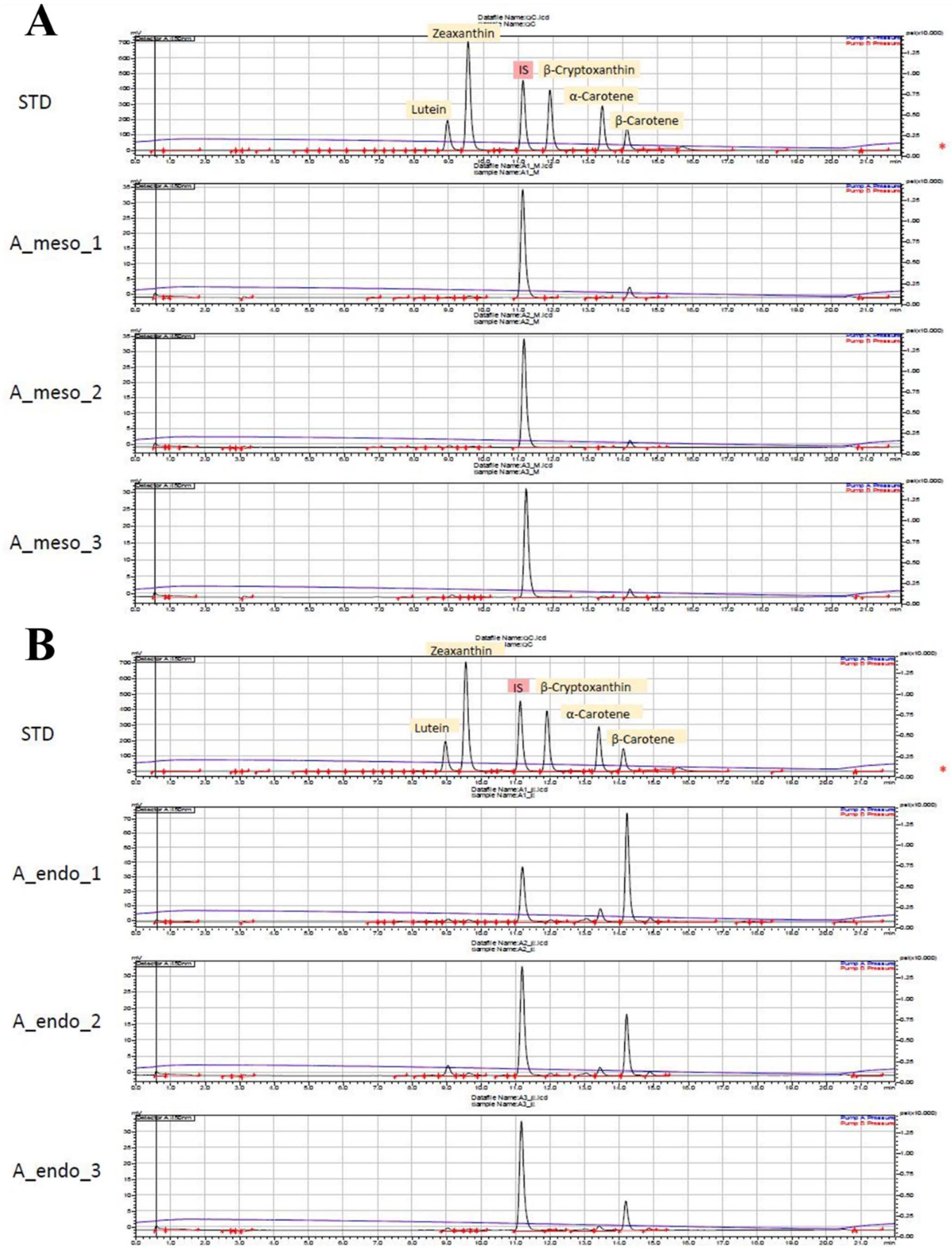

Supplementary Figure 5. LC-DAD spectrum of standard and CS-A. (**A**) Reference standard peak and peaks analogous to three mesocarp samples and reference standard peak. (**B**) Peaks analogous to three endocarp samples

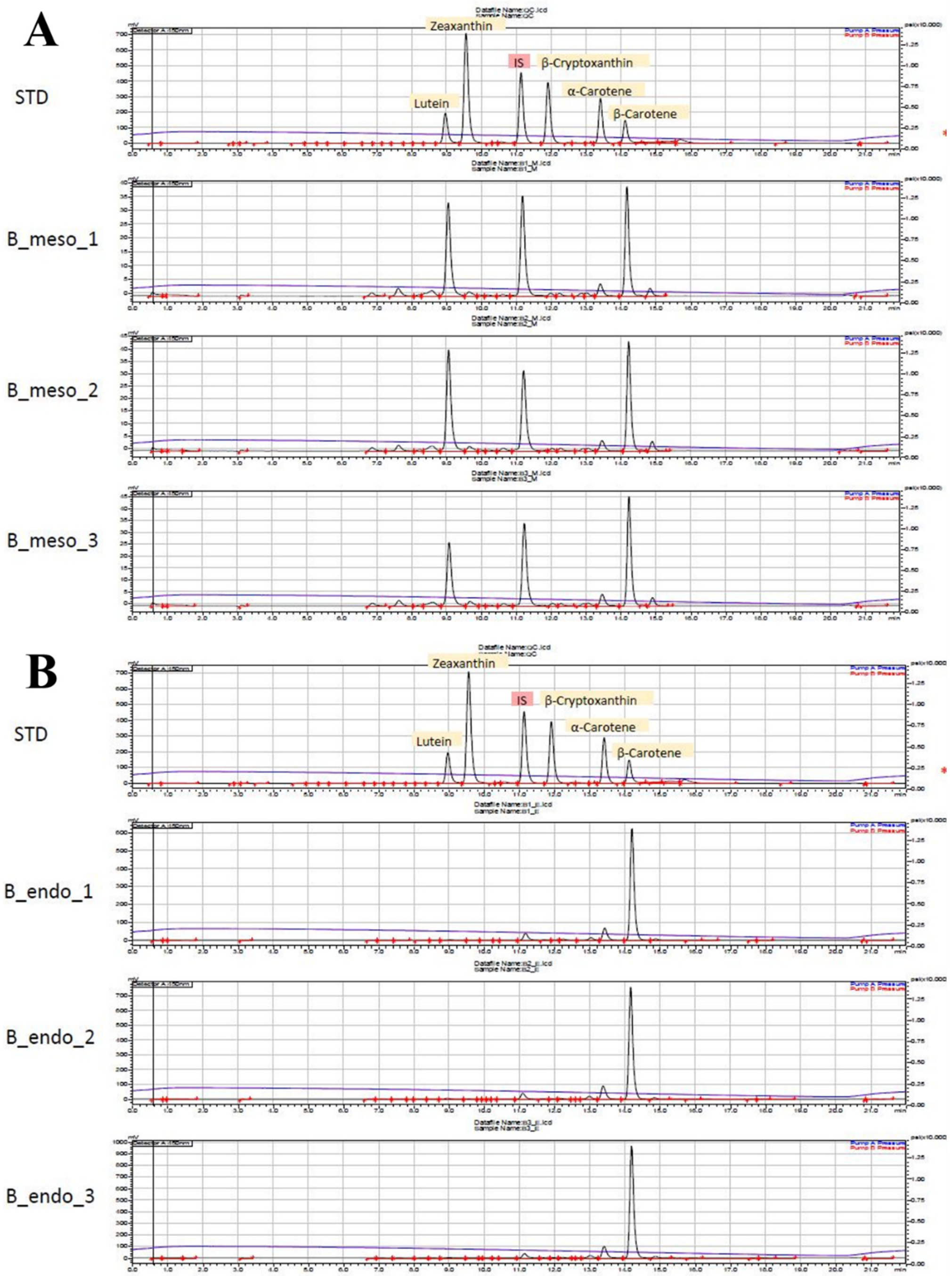

Supplementary Figure 6. LC-DAD spectrum of standard and CS-B. (**A**) Reference standard peak and peaks analogous to three mesocarp samples. (**B**) Reference standard peak and peaks analogous to three endocarp samples.

1.2 Supplementary Tables

Supplementary Table 1. Primers used for HRM analysis

Gene Id	Position	Variation type	Primer name	Primer sequence (5'-3')
CsaV3_4G000740	421,165	SNP	CsaLCYB-HRM-1F	ATACACTTTCCCCCCATGTTTT
			CsaLCYB-HRM-1R	AAGCTTAAGGGGTTGAGGTTTG
	422,742	InDel (8 bp)	CsaLCYB-HRM-2F	GGAGGGTCTTTTGTCTTCT
			CsaLCYB-HRM-2R	TATAAACTAAATTATAATAACCAACTATATACATG
CsaV3_6G040750	23,615,309	InDel (1bp)	CsaOR-HRM-3F	TTCAATCAGATTATAGTGGCCT
			CsaOR-HRM-3R	TTAGTCAAATGGGTCGAATCT
	23,619,716	SNP	CsaOR-HRM-2F	TCTTCGCTCCTTCCTCTC
			CsaOR-HRM-2R	AAACTAACCTGGAAGTGTTATCACT
	23,619,823	SNP	CsaOR-HRM1F	CATTAGCCCTATCCCCTTCT
			CsaOR-HRM1R	CTATAATCGATGGACAAGAAAATTG
	23,619,827	SNP	CsaOR-HRM1F	CATTAGCCCTATCCCCTTCT
			CsaOR-HRM1R	CTATAATCGATGGACAAGAAAATTG

Supplementary Table 2. Primers used for Real-time PCR

Gene id	Given name	Primer name	Sequence (5'- 3')
CsaV3_6G040750	*Or*	Csa6G040750-qPCR-F	GATGTTCTTCCAGTGGCGTC
		Csa6G040750-qPCR-R	GTGCTCACTTGCCATCAACA
Csa_6G484600	*Actin*	CsActin2-qPCR-F	ATTCTTGCATCTCTAAGTACCTTCC
		CsActin2-qPCR-R	CCAACTAAAGGGAAATAACTCACC

Supplementary Table 3. Summary of linkage map generated using 160 SNPs

Linkage group	Chromosome	Number of SNPs
1	7	1
2	7	10
3	6	3
4	5	24
5	4	1
6	4	3
7	4	13
8	4	1
9	3	1
10	3	3
11	3	4
12	3	26
13	2	1
14	2	1
15	2	21
16	1	3
17	1	9
18	1	4
19	6	31
Total		160

Supplementary Table 4. Distribution pattern of SNPs in CS-A and CS-B relative to the 'Chinese Long v3' reference genome

**Chromosome**	**CS-A**	**CS-B**
1	55,374	105,579
2	42,025	140,973
3	90,534	207,020
4	38,066	135,405
5	43,237	94,076
6	60,402	173,006
7	38,654	92,895
Others	2,285	2,861
Total	370,577	951,815
